# A Review of micro RNAs changes in T2DM in animals and humans

**DOI:** 10.1111/1753-0407.13431

**Published:** 2023-06-17

**Authors:** Mohammad Reza Afsharmanesh, Zeinab Mohammadi, Azad Reza Mansourian, Seyyed Mehdi Jafari

**Affiliations:** ^1^ Metabolic Disorders Research Center Golestan University of Medical Sciences Gorgan Iran; ^2^ Department of Biochemistry and Biophysics, School of Medicine Golestan University of Medical Sciences Gorgan Iran

**Keywords:** inflammation, insulin resistance, microRNA, type 2 diabetes mellitus, 炎症, 2型糖尿病, miRNA, 胰岛素抵抗

## Abstract

Type 2 diabetes mellitus (T2DM) and its associated complications have become a crucial public health concern in the world. According to the literature, chronic inflammation and the progression of T2DM have a close relationship. Accumulated evidence suggests that inflammation enhances the insulin secretion lost by islets of Langerhans and the resistance of target tissues to insulin action, which are two critical features in T2DM development. Based on recently highlighted research that plasma concentration of inflammatory mediators such as tumor necrosis factor α and interleukin‐6 are elevated in insulin‐resistant and T2DM, and it raises novel question marks about the processes causing inflammation in both situations. Over the past few decades, microRNAs (miRNAs), a class of short, noncoding RNA molecules, have been discovered to be involved in the regulation of inflammation, insulin resistance, and T2DM pathology. These noncoding RNAs are specifically comprised of RNA‐induced silencing complexes and regulate the expression of specific protein‐coding genes through various mechanisms. There is extending evidence that describes the expression profile of a special class of miRNA molecules altered during T2DM development. These modifications can be observed as potential biomarkers for the diagnosis of T2DM and related diseases. In this review study, after reviewing the possible mechanisms involved in T2DM pathophysiology, we update recent information on the miRNA roles in T2DM, inflammation, and insulin resistance.

## Graphical abstract

**Highlights**

- MicroRNAs (miRNAs) are overexpressed in inflammation, insulin resistance, and type 2 diabetes mellitus (T2DM).
- Many types of miRNAs are changed during the T2DM progression.
- miRNAs have a potential role as a biomarker in T2DM diagnosis.

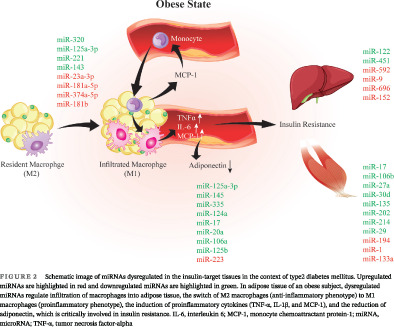

## 摘要

2型糖尿病(T2DM)及其相关并发症已成为全球重要的公共卫生问题。根据现有文献，慢性炎症与2型糖尿病的进展有密切关系。越来越多的证据表明，炎症增强了胰岛素分泌功能的丧失和靶组织对胰岛素作用的抵抗，这是2型糖尿病发展的两个关键特征。最近的研究表明，胰岛素抵抗和2型糖尿病患者血浆中炎症介质如肿瘤坏死因子α (TNF‐α)和白细胞介素6 (IL‐6)的浓度升高，这对这两种情况下引起炎症的过程提出了新的疑问。miRNAs是一类短链非编码RNA分子，在过去的几十年里被发现参与调节炎症、胰岛素抵抗和2型糖尿病的病理过程。这些非编码RNA特异性地由RNA诱导沉默复合物组成，并通过各种机制调节特定蛋白编码基因的表达。越来越多的证据表明，在T2DM发展过程中，一类特殊miRNA分子的表达谱发生了改变。这些修饰可作为诊断T2DM及相关疾病的潜在生物标志物。在了解了2型糖尿病可能的病理生理机制后，本文就miRNA在2型糖尿病、炎症和胰岛素抵抗中的作用进行综述。

## 1 INTRODUCTION

In 2017, diabetes mellitus (DM) has become a global challenge to human health, which affects 451 million individuals. In this way, the prevalence of diabetes is dramatically increased in all parts of the world. Dramatically, the prevalence of diabetes increased in many countries of the world. The International Diabetes Federation predicts that the number of people affected by diabetes will rise to approximately 693 million by the year 2045.^1^ Type 1 diabetes mellitus (T1DM) and type 2 diabetes mellitus (T2DM) are two major pathological classifications of DM. T1DM is an autoimmune disorder characterized by absolute insulin deficiency production due to the dysfunction of insulin‐producing β‐cells of the pancreas. Therefore, after destruction of over 90% of the β‐cells, a hyperglycemia situation emerges.^2^

As well, T2DM accounts for approximately 90%–95% of diabetes patients and is strongly associated with insulin resistance and disruption of blood sugar homeostasis.^3^ In diabetes, chronic hyperglycemia leads to microvascular complications such as retinopathy, neuropathy, and nephropathy or macrovascular complications, which is the cause of atherosclerotic disease.^4^ Accumulated evidence exists to clarify the cellular and molecular mechanisms that are frequently involved in the pathogenesis of T2DM. However, there is growing evidence that low‐severity chronic inflammation caused by obesity is the primary cause of the T2DM initiation and development.^5^ Recently, several studies illustrated that microRNAs (miRNAs) are involved in the onset and development of T2DM, by focusing on miRNA involvement in the regulation of cell function, insulin secretion, and the insulin signaling pathways in target tissues.^6^ In mid‐1993, the earliest miRNA was identified. Moreover, more than 2000 microRNAs have been classified so far in humans alone.^7^ Several studies revealed that miRNA profile expression has been altered in T2DM. Hence, this evidence suggested the potential of miRNAs as a biomarker for diagnosis and treatment.^8^, ^9^ Therefore, the present review aims to highlight the recent findings and important challenges associated with miRNA studies in diabetes, with a particular focus on critical factors contributing to T2DM development, such as obesity, inflammation, and insulin resistance.

## 2 miRNAs' SYNTHESIS AND FUNCTION

miRNAs are a large family of short noncoding RNA sequences, 21–22 nucleotides in length, that act as modulators of gene expression. These small molecules are distributed throughout the genome and contain both non‐coding (in introns) and protein‐coding regions (in exons).^10^ First, miRNAs in primary (pri‐miRNA) form, immature molecules, are synthesized in the nucleus, which undergo a series of processing steps in the nucleus by Drosha and then in the cytoplasm by the Dicer enzyme to become mature miRNAs. Upon maturation, single‐stranded molecules are specifically incorporated into RNA‐induced silencing complexes. They can bind to 3′‐untranslated region (3′UTR) of their target mRNA sequences, leading to mRNA silencing or degradation.^11^ Lin‐4, the first miRNA, was identified in the nematode *Caenorhabditis elegans* (*C. elegans*) in 1993. Afterward, various miRNA molecules were discovered in vertebrates and plants.^12^ Over 2654 mature miRNAs have been identified in the human genome, which is robustly involved in the modulation of 14 300 genes.^13^ Besides, miRNAs are multifunctional molecules because every single of them could broadly modify different target mRNAs in a cell type, and different miRNAs can affect each mRNA.

Additionally, miRNAs regulate many different cellular events including cell growth, cell death, proliferation, and differentiation.^14^, ^15^ These molecules have emerged in the last decade as regulatory keys of metabolic homeostasis. Therefore, disruption of miRNA expression might lead to T2DM initiation and development.^16^

## 3 microRNAs' ROLES IN T2DM DEVELOPMENT

As mentioned, miRNAs play regulatory roles in many biological events and show an important correlation with metabolic diseases such as obesity and T2DM. Mersey and colleagues provided the first evidence of miR‐29 b's role in human metabolic pathway regulation.^16^ miRNA's role in T2DM development first was defined by the discovery of miR‐375's role in the regulation of insulin secretion.^17^ Recent studies have demonstrated that miRNAs are also involved in pancreatic cell and insulin function and insulin production in target tissues such as skeletal muscle, fat, and liver.^18^

## 4 ROLE OF miRNAs IN β‐CELL SURVIVAL

The β‐cells critical role in glucose homeostasis was illustrated through DM development process by losing β‐cells. β‐cells functions are regulated at different levels, and miRNA molecules establish important key points for cellular response integration.^19^ The increased levels of metabolites, such as fatty acids and glucose, in the bloodstream during T2DM development, may also contribute to the regulation of miRNA levels. The critical role of miRNAs in the development and function of β‐cells was emphasized by examining Dicer1 knockout animals. Dicer1 deletion or knockdown significantly reduces cell mass, resulting in a functional defect.^20^ Although very few of the miRNAs are tissue specific, many of them can be localized in the endocrine sections of the pancreas. The most common miRNA that is detected in human islet cells, miR‐375, was published, as the first publication on miRNA, in the past decade. Poy et al showed that about 10% of the total miRNA in pancreatic cells belonged to miR375.^21^ This miRNA is required to regulate genes involved in normal β‐cells mass maintenance, insulin secretion, and glucose homeostasis.^22^, ^23^ The miR375 knockout mice (375KO) exhibit reduced β‐cells mass, impaired cell proliferation, and a progressive hyperglycemic phenotype.^24^ Ouamari et al provided evidence that miR‐375 targets phosphoinositide‐dependent protein kinase‐1 (PDK1), a key intermediate of phosphoinositide 3‐kinase (PI3K) cascade, and reduces its expression in both the mRNA and protein levels. By PDK1 targeting, miR‐375 reduces the phosphorylation of Akt and glycogen synthase kinase‐3 (GSK3), two downstream targets of PDK1. This signaling cascade is important in β‐cell mass.^25^

Additionally, the miRNA‐200 family members such as miR‐200a, miR‐200b, miR‐200c, miR‐141, and miR‐429 are other abundant miRNAs that are expressed in pancreatic islet cells and regulate β‐cell survival. Belgardt et al revealed that overexpression of the miR‐200 gene family in mice promotes β‐cell apoptosis and the T2DM state. mir‐200 positively modulates Trp53 activation, thereby generating a proapoptotic gene expression signature in islets from diabetic mice.^26^

In diabetics, miRNAs of the miR‐29 family are expressed in a variety of tissues, including liver, kidney, cells, adipose, and skeletal muscle tissue. Multiple evidence has identified the miR29 family, including miR29a, miR29b1, miR29b2 and miR29c, as key modulators of various functions in β‐cells.^27^, ^28^ It has been determined that hallmarks of DM, such as hyperglycemia and inflammation, result in miR‐29 family miRNAs upregulation. Roggli et al have been shown that exposure of pancreatic β‐cells to proinflammatory cytokines increased the expression of miR‐29 family members and thus cell death. An animal model study association between inflammation and miR‐29 increased levels were revealed in β‐cells. A study by Roggli et al on animal models revealed an association between inflammation and overexpression of miR‐29 in β‐cells.^29^ Sun et al showed that miR‐29 promotes circulating monocyte recruitment and inflammation in β‐cells via a tumor necrosis factor alpha (TNF‐α) receptor‐associated factor 3‐dependent signaling pathway.^30^ miR‐486‐5p overexpression increases pancreatic β‐cell proliferation, insulin sensitivity, and suppresses apoptosis in a pancreatic β‐cell line by targeting phosphatase and tensin homolog (PTEN) and forkhead O1 box (FOXO1). Tian et al indicated the miR‐486‐5p expression in the peripheral blood of T2DM patients is lower than in healthy subjects.^31^

β‐cell mass and insulin synthesis are stimulated by nutrition and growth factors via multiple intracellular signaling pathways including insulin receptor substrate 1 (IRS‐1)‐PI3K‐Akt, Janus kinase (JAK)/signal transducer and activator of transcription (STAT), and mitogen‐activated protein kinase. Various studies suggests that the suppressor of cytokine signaling 3 (SOCS3) is associated with the suppression of insulin synthesis and secretion through inactivation of the JAK/STAT pathway.^32^ Recently, studies have indicated that miR‐19a‐3p promotes pancreatic β‐cell proliferation and insulin secretion and suppresses pancreatic β‐cells apoptosis by targeting SOCS3 expression. In the diabetic state, SOCS3 levels were elevated and inversely associated with miR‐19a‐3p levels, suggesting that suppression of miR‐19a‐3p leads to overexpression of SOCS3, contributing to β‐cells dysfunction.^33^ miR‐185 is another miRNA that directly targets the SOCS3 gene by binding to its 3′‐UTR to prevent β‐cell dysfunction in diabetes conditions. Bao et al found an inverse correlation between SOCS3 and miR‐185 levels in plasma samples of diabetic patients. In murine pancreatic β‐cells transfected with miR‐185 mimics, miR‐185 effectively suppressed the SOCS3 mRNA and protein expression levels and enhanced insulin secretion and total insulin content.^34^ Mohan et al also reported that the imbalance between miR‐483 and its targets, such as SOCS3, may play a vital role in the diabetes pathogenesis.^35^

Yu et al. indicated miR‐125b‐5p promotes islet cells proliferation and apoptosis inhibition in T2DM mice by repressing c‐Jun NH2‐terminal kinase (JNK) signaling cascade by targeting Disheveled antagonist Dapper1 (DACT1) in islet cells. DACT1 is a functional candidate gene associated with embryonic development and lipid metabolism, affecting many aspects of growth such as survival rate, energy intake, and body weight in mammals.^36^ Several clinical trial studies indicated miRNAs expression levels were changed in pancreatic islets in T2DM patients compared to individuals without diabetes. Locke et al assessed the global profile of islet miRNA expression in 11 individuals with T2DM and 9 controls employing global Taq man arrays. They illustrated that miR‐187 expression upregulated in the islets of Langerhans in T2DM patients compared to the matched controls. The gene‐coding homeodomain‐interacting protein kinase‐3 is an important target of miR‐187, which has a vital role in insulin secretion. Therefore, the overexpression of miR‐187 in diabetic patients associated with decreasing the insulin secretion from Langerhans islet.^37^

## 5 ROLE OF miRNAs IN INSULIN PRODUCTION AND SECRETION

### 5.1 Roles in β‐cells function

In 1916, Schafer et al suggested that insulin is a glucose‐lowering hormone that is synthesized and secreted from pancreatic islet cells.^38^ Insulin hormone is synthesized in the β‐cells of the pancreas as a 110‐amino acid precursor called pre‐pro‐insulin. During the maturation process, pre‐pro‐insulin undergoes conformational adjustment in the endoplasmic reticulum (ER) to produce a pro‐insulin structure. Then, pro‐insulin is transported from the ER to the Golgi apparatus and converted into C‐peptide and native insulin. Human mature insulin is a polypeptide hormone 51 amino acids in length and with a molecular weight of 5.8 kDa.^39^ Although many signals affect insulin production and secretion, glucose metabolism is the most important event that controls the transcription and translation of insulin genes. After a meal, the increased circulating glucose levels lead to increased glucose uptake via β‐cells through glucose transporters (GLUTs). GLUT1 is the main glucose transporter in human pancreatic β‐cells.^40^ In the β‐cells, glucose is metabolized in the glycolytic pathway, resulting in the production of ATP and an increase in the ATP:ADP ratio. These events result in ATP‐sensitive potassium channel closure and subsequent cell membrane depolarization, resulting in calcium influx from the extracellular space through a voltage‐gated calcium channel. The increased level of intracellular calcium leads to fusion of the secretory vesicles with the plasma membrane of β‐cells and exocytosis of insulin.^41^ Recent studies demonstrated that many types of miRNAs can target insulin synthesis, ATP:ADP ratio, β‐cell membrane depolarization, vesicle fusion, and exocytic exocytosis processes. In pancreatic β‐cells, uncoupling protein 2 (UCP2) promote mitochondrial proton leakage and the reduction of ATP levels and finally glucose‐stimulated insulin secretion. Zhang et al indicated that UCP2‐deficient mice had higher levels of ATP and insulin secretion which is suggested UCP2 negatively controls the insulin secretion process.^42^ It was revealed that miR‐15a directly targets the UCP2 mRNA in β‐cells and positively regulates insulin biosynthesis and secretion processes. Hennessy et al showed that prolonged exposure of murine insulinoma (MIN6) cells to glucose downregulates miR‐15a, resulting in an increment in UCP2 levels and a decrease in insulin secretion.^43^

miR‐375 overexpression suppressed insulin secretion from pancreatic β‐cells, whereas the inhibition of miR‐375 increased insulin exocytosis. A previous study reported that miR‐375 targets the 3'‐UTR of myotrophin (Mtpn) mRNA to block glucose‐stimulated insulin exocytosis from pancreatic β‐cells.^44^ Mtpn is involved in the process of cytoskeletal remodeling through the depolymerization of actin filaments, allowing for the fusion of secretory insulin vesicles with the β‐cell membrane. In addition, Mtpn overexpresses the nuclear factor kappa B (NF‐κB), which can subsequently stimulate the expression of genes involved in transport of insulin vesicles to the cell membrane.^45^

Several studies suggested miR‐200c is one of the most important miRNAsin the β‐cell, playing a critical role in insulin secretion and also β‐cell apoptosis in human islets by regulation of numerous key gene expressions such as juxtaposed with another zinc finger protein 1 (JAZF1),^26^ zinc finger E‐box binding homeobox 1,^46^ and Ets variant5 (ETV5).^47^ Ofori et al identified miR‐200c as a miRNA significantly overexpressed in human islets from T2DM donors. miR‐200c directly targeted ETV5 in human islets, resulting in decreased insulin secretion, a key phenotype of T2DM patients. ETV5 induces insulin exocytosis in human islets through positive regulation of synaptosome‐associated protein 25 kDa (SNAP‐25) and vesicle associated membrane protein 2 expression. Jazf1 is another target gene of miR‐200c identified in human islets from T2DM donors.^47^ Recently, it has been suggested that Jazf1 knockdown in mice leads to increased endoplasmic reticulum (ER) stress and thereby propagating susceptibility to β‐cell apoptosis.^26^ However, Ofori et al suggested that inhibition of miR‐200c by LNA200c might improve insulin secretion in islets of T2DM donors.^47^

**FIGURE 1:**
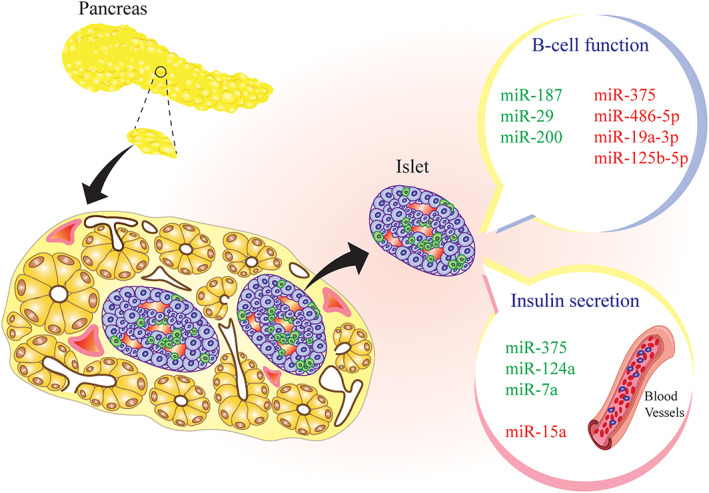
Schematic image of the miRNAs dysregulated in pancreatic β‐cells. Upregulated miRNAs are highlighted in red, and downregulated miRNAs are highlighted in green. miRNA, microRNA.

Interestingly, it was declared that miR‐124a affect sthe exocytosis and insulin secretion in MIN6 cells. Furthermore, in pancreatic islets with T2DM the gene expression level upregulated. Subsequently, this situation led to disrupting the insulin secretion trough inhibiting the expression of the glucose regulatory sensor genes such as Forkhead box A2 (FOXA2), which is involved in insulin secretion exocytosis machine.^48^ In the β‐cell line MIN6B1, miR‐124a downregulates Rab27a and upregulates rabphilin 3A (Rab3A), SNAP‐25, and synapsin‐1A (syn‐1) expression. Rab27a is a GTPase selectively enriched in endocrine cells and involved in vesicle transfer to the cell membrane. Therefore, downregulation of Rab27a by miR‐124a alleviates the secretory effects of pancreatic β‐cells in response to high glucose conditions.^49^ Using a transgenic mouse, Latreille et al illustrated that miR‐7a overexpression leads to impaired insulin secretion by directly targeting genes contributed to insulin granule fusion and activation of SNARE complex.^50^ A recent study showed that miR‐7 can regulate the glucagon‐like peptide‐1 (GLP‐1)/β‐Arrestin1 axis in pancreatic β‐cells. GLP‐1 has been revealed to stimulate glucose‐induced insulin release by binding to the GLP‐1 receptor on β cells. β‐arrestin 1 plays a significant role in desensitizing this receptor in β‐cells. Matarese et al. reported that miR‐7 directly targets β‐arrestin 1 and is key to stimulating GLP‐1‐mediated insulin secretion.^51^ It is briefly demonstrated in Figure 1.

## 6 ROLE OF miRNAs IN INSULIN RESISTANCE

More and more studies have shown that miRNAs are also involved in the manifestation of insulin resistance. The association between miRNAs and inflammation associated with obesity, the main mechanism of insulin resistance, is discussed next.

## 7 ROLE OF miRNAs IN ADIPOSE TISSUE FUNCTION AND INSULIN RESISTANCE

Obesity is a chronic, low‐grade inflammatory condition closely associated with insulin resistance and T2DM. The obese patients with T2DM often have the abnormal lipid metabolism. In obesity condition, the increased serum level of free fatty acids (FFA) alleviated the insulin‐induced glucose uptake by cells and also insulin resistance phenomenon.^52^ Also, FFAs affect the activation of intermediates in insulin signaling. They primarily decrease IRS‐1‐associated PI3K activity, which can disrupt other downstream insulin signal transduction cascades.^53^ Due to the vital role of miRNAs in controlling gene expression in cells, miRNA dysregulation alters adipocyte functions and is implicated in obesity and insulin resistance. The importance and role of miRNAs in adipocyte biology are made clear by the pioneering research by Esau and co‐workers in 2004. Their results showed that miR‐143 inhibits preadipocyte differentiation into adipocytes by interfering with the ERK5 signaling pathway. Inhibition of miR‐143 molecule results in the down‐egulation of adipocytes specific genes such as adipocyte protein 2 (aP2), GLUT‐4, peroxisome proliferator‐activated receptor gamma (PPAR‐γ)‐2, and hormone‐sensitive lipase.^54^ In another study, Ling et al observed that adipocytes increase expression of miR‐320 in an insulin resistance state. They also revealed that miR‐320 suppresses the expression of the p85 subunit of the PI3K enzyme, resulting in a blockage of the insulin hormone signaling pathway.^55^ Regarding the role of miR‐125a‐3p in obesity, Yeh et al reported that high levels of miR‐125a‐3p in obese subjects are positively associated with the expression of proinflammatory genes and negatively associated with the insulin receptor and the further downstream target of PI3K, indicating the role of this miRNA in the regulation of the insulin signaling cascade.^56^ MiR‐374a‐5p is one of the miRNAs that is upregulated in healthy obese individuals. It is associated with the downregulation of proinflammatory cytokines, including the C–C motif chemokine ligand 2 (CCL2), resulting in low inflammation and protecting against insulin resistance and T2DM.^57^ A study by Sun et al showed that the expression of miR‐181b was reduced in adipose tissue of obese mice. This miRNA plays an important role in regulating endothelial function in adipose tissue, improving glucose homeostasis and insulin sensitivity.^58^

The recent studies illustrated that the gene expression of the seven miRNAs, such as let‐7f‐5p, miR‐335‐5p, miR‐15b‐5p, miR‐7‐5p, let‐7i‐5p, miR‐205‐5p, and miR‐320c, was altered in obese patients from pre‐ to postbariatric surgery and with weight loss Incredibly, these miRNAs target genes that contribute to insulin resistance and functional pathways related to T2DM.^59^ A worldwide meta‐analysis of human studies that identified the importance of circulating miRNAs in the diagnosis of obesity and T2DM reported that miR‐142‐3p and miR‐222 were significantly upregulated in obese and T2DM patients.^60^ Bartolomé et al found that miR‐23a‐3p and miR‐181a‐5p were downregulated in adipose tissue in obese individuals and inversely associated with insulin resistance. These miRNAs can inhibit TNF‐α‐stimulated insulin resistance in adipocytes by modulating the expression of key molecules of the insulin signaling pathway, such as PTEN and p70S6K.^61^ Meerson and colleagues provide data showing that miR‐221 was overexpressed in obese individuals and may be involved in the development of T2DM by directly downregulating adiponectin receptor 1 (ADIPOR1) mRNA expression.^62^ ADIPOR1 is a receptor for adiponectin and triggers a signal transduction pathway that enhances insulin sensitivity and is downregulated in obesity‐induced insulin resistance and T2DM.^63^ It was reported that expression of miR‐592 was alleviated in the liver of obese mice and humans, leading to hepatic triglyceride accumulation, enhanced gluconeogenesis, hyperglycemia, and insulin resistance. Overexpression of this miRNA in obese mice ameliorates metabolism of glucose in hepatic cells through binding to the 3′‐UTR of FOXO1.^64^

## 8 MicroRNAs AND OBESITY‐RELATED INFLAMMATION

In the early 1990s, the first evidence to determine the inflammatory origin of obesity was raised from animal and human research. In these studies, obese rodents and human adipose tissue have demonstrated higher levels of pro‐inflammatory cytokines such as TNF‐α, which can promote insulin resistance by inactivating the IRS‐1 molecule.^65^, ^66^ Currently, the new evidence has confirmed and extended the early findings. Many studies confirm the vital role of immune cells in including local inflammation in adipose tissue that causes insulin resistance. A proinflammatory state caused by obesity is defined by T cells and macrophages infiltration and activation which release cytokines and chemokines contributing to the dysregulation of fatty acid metabolism in adipocytes.^67^ Various miRNAs regulate macrophage infiltration into adipose tissue and switching from M2 macrophages (anti‐inflammatory phenotype) to M1 macrophages (proinflammatory phenotype), thereby promoting chronic inflammation and insulin resistance in obese. M1 macrophages secrete an increasing number of pro‐inflammatory cytokines such as TNF‐α, interleukin (IL)‐1β, and IL‐23, which are critically involved in insulin resistance and T2DM.^68^ Much evidence confirms the crucial roles of miRNAs in macrophage infiltration and activation regulation via bind to the regulatory key genes. Nakamachi et al emphasized the miR‐124a role in inducing CCL2 expression, thereby enhancing adhesion of monocyte to the vessel wall.^69^ In another study, Zhu et al showed that miR‐17, miR‐20a, and miR‐106a promote macrophage infiltration and proinflammatory cytokine secretion by commonly inhibiting signal‐regulatory protein α expression.^70^ It has been clarified miR‐223 plays a critical role in M2 phenotype maintaining in adipose tissue.

**FIGURE 2:**
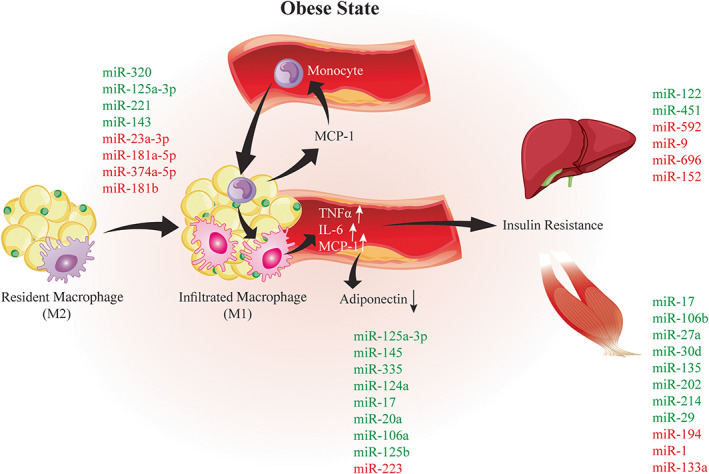
Schematic image of miRNAs dysregulated in the insulin‐target tissues in the context of type2 diabetes mellitus. Upregulated miRNAs are highlighted in red and downregulated miRNAs are highlighted in green. In adipose tissue of an obese subject, dysregulated miRNAs regulate infiltration of macrophages into adipose tissue, the switch of M2 macrophages (anti‐inflammatory phenotype) to M1 macrophages (proinflammatory phenotype), the induction of proinflammatory cytokines (TNF‐α, IL‐1β, and MCP‐1), and the reduction of adiponectin, which is critically involved in insulin resistance. IL‐6, interleukin 6; MCP‐1, monocyte chemoattractant protein‐1; miRNA, microRNA; TNF‐α, tumor necrosis factor‐alpha

Furthermore, the inhibition of miR‐223 leads to switch of M2 into M1 macrophages.^71^ Also, a previous report illustrated that miR‐125b suppressed interferon regulatory factor 4 expression in macrophages and modulated the number of pro‐inflammatory phenotypes of macrophages.^72^ Lorente‐Cebrián et al pointed out that miR‐145 by the release of TNF‐α from adipocytes induces the manifestation of chronic inflammation. The production of TNF‐α is accelerated by activating the NF‐кB signaling pathway.^73^ In addition to, MiR‐145 attenuates the TNF‐α‐converting enzyme expression, also called A disintegrin and metalloprotease‐17 (ADAM17), leading to increase binding the fraction of TNF‐α to the cell membrane, which is the most active form of this cytokine.^74^ Zhu et al revealed miR‐335 expression is increased by TNF‐α, IL‐6, leptin, and resistin in human mature adipocytes.^75^ In the obesity state, adipose tissue releases various proinflammatory cytokines, such as TNF‐α and IL‐6, and adipokines, such as resistin and leptin, which promote pathways affecting insulin resistance.^76^ Peng et al confirmed that miR‐377 intensify obesity‐induced inflammation and insulin resistance, by binding to 3′‐UTR of Sirtuin‐1 (Sirt1) mRNA.^77^ We illustrated this briefly in Figure 2.

## 9 ROLE OF miRNAs IN LIVER METABOLISM IN INSULIN RESISTANCE

Liver tissue plays the energy metabolism as it contributes significantly to the metabolism of glucose, amino acids, and lipoproteins. miR‐122 is the most abundant miRNA in the liver, which is involved in hepatic cholesterol metabolism.^78^ Two studies have indicated that miR‐122 inhibition leads to a significant decrease in plasma cholesterol level.^78^, ^79^

Several miRNAs affect hepatic insulin action and glucose homeostasis.^80^, ^81^ Recent studies indicated that miRNA‐9, miR‐146b, miRNA‐451, and miRNA‐696 play a crucial role in hepatic gluconeogenesis. MiR‐9 reduce the hepatic gluconeogenesis and glucose production by directly targeting the FOXO1 transcription factor. In hepatocytes, FOXO1 induces the gluconeogenic enzymes transcription such as glucose‐6‐phosphatase (G6Pase) and phosphoenolpyruvate carboxy kinase (PEPCK), promoting hepatic glucose production.^82^ Yan et al illustrated that miR‐9‐3 promoter hypermethylation and miRNA‐9 expression was diminished in the obese mice liver. Downregulation of miRNA‐9 leads to FOXO1 upregulation in the obese mice livers and hepatic gluconeogenesis activation.^83^ Previously, the miR‐146b/Sirt1‐FOXO1 cascade's contribution to regulation of adipogenesis and liver glycogenesis has been confirmed. Upregulation of miR‐146b decreases Sirt1 gene expression and subsequent acetylation of FOXO1 in diabetic mice liver and HepG2 cells, in response to the insulin resistance induction and the suppression of hepatic glycogenesis.^83^ Based on the Wang et al report Sirt1 deficiency in the hepatic mouse resulted in overproduction of hepatic glucose, chronic hyperglycemia, and subsequently enhanced reactive oxygen species (ROS). The ROS overproduction disrupted mTOR/Akt signaling in the insulin‐sensitive target organs, causing insulin resistance. According to the mentioned, these findings suggested that antioxidant treatment might reverse progressive insulin resistance and improve T2DM in older human populations.^84^ Zhuo et al illustrated that miR‐451 was remarkably upregulated in the diabetic mice hepatic tissue. They found that miR‐451 regulates hepatic gluconeogenesis, inhibits hyperglycemia, and improves glucose tolerance by targeting the AKT‐FOXO1‐PEPCK/G6Pase pathway.^81^ Fang et al confirmed miRNA‐696 has been association with peroxisome proliferator‐activated receptor gamma coactivator‐1 alpha (PGC‐1α) levels in diabetes. They proved this miRNA plays a critical role in hepatic gluconeogenesis and insulin resistance. In hepatocytes, miRNA‐696 binds directly to a specific region within the 3′‐UTR of PGC‐1α, and suppressing PEPCK translation, a rate‐limiting enzyme of gluconeogenesis induced by PGC‐1α.^85^ MiR‐152 is one of the other miRNAs that regulate hepatic glycogenesis trough PTEN regulation. However, miR‐152 expression is reduced in the liver tissue of diabetic mice, which leading to impaired hepatocytes glycogenesis.^86^ It is illustrated in Figure 2.

## 10 ROLE OF MICRO RNAs ON SKELETAL MUSCLE INSULIN SENSITIVITY

The disruption of glucose uptake and insulin signaling cascade has been implicated in the insulin resistance phenomenon in skeletal muscle cells in several studies. Some miRNAs have been likened to insulin resistance in skeletal muscle and have been shown to be dysregulated in invivo and in vitro studies. miRNA expression increases in skeletal muscle in response to certain miRNAs such as miR‐17, miR‐29a, miR‐214, miR‐135a, miR‐106b, and miR‐27a, while the expression of others such as miR‐1, miR‐133a, and miR‐194, decreases.^87^ Xiao et al showed that miRNA‐17 is the most abundant expressed miRNA in the skeletal muscle cells of T2DM rats. Loss‐ and gain‐of‐function studies revealed miR‐17 overexpression disrupted glucose metabolism in L6 skeletal muscle cells. This study demonstrated that miR‐17, as a novel mechanism, directly suppresses GUT4 in the presence of insulin resistance.^88^ Zhou et al indicated that miR‐106b, miR‐27a, and miR‐30d play the critical roles in glucose homeostasis regulation and insulin resistance in the insulin‐resistant cells and skeletal muscle in diabetic rats. They suggested that these miRNAs expression increased in insulin‐resistant L6 cells that are led to diminished glucose uptake, GLUT4 expression and PI3K regulatory subunit beta.^89^ In a recent study, conducted by Honardoost et al confirmed that the upregulation of miR‐135, miR‐202, and miR‐214 in muscle cell lines resulted in reduced glucose uptake by insulin pathway, insulin resistance induction, and development of T2DM phenotype.^90^ MiR‐27a is an important adipocyte‐derived miRNA that secreted into the bloodstream and play the critical role in skeletal insulin resistance. It can induce insulin resistance in skeletal muscle through targeting PPAR‐γ expression and its downstream genes involved in obesity development.^91^ Latouche et al revealed that miR‐194 expression is downregulated in skeletal muscle cells in prediabetic and diabetic rats and humans, which leading to glucose uptake and glycogen synthesis elevation. In L6 skeletal muscle cells, miR‐194 is implicated in numerous aspects of glucose metabolism, including uptake, glycolysis, glycogenesis, and mitochondrial oxidative phosphorylation, which involve AKT and GSK3 signaling pathways.^92^, ^93^ It is shown in Figure 2.

## 11 CIRCULATING miRNAs AS POTENTIAL BIOMARKERS IN PREDICTING T2DM

In the recent decade, several studies have uncovered many dysregulated circulating miRNAs in the serum/plasma of prediabetic and diabetic patients. Moreover, the case–control studies demonstrated the miRNAs profiles may be altered in response to the glucose homeostasis disruption.

The case–control studies on healthy, prediabetic, and diabetic patients illustrated that the miRNA profile have a significantly alternation before the serum level of glucose homeostasis disrupted. In a case–control study, Zeinali et al suggested that miR‐122, miR‐126‐3p, and miR‐146a may be potentially associated with inflammatory processes and could contribute to the T2DM pathogenesis. Chronic inflammation in insulin‐responsive target tissues is accompanied by ROS levels elevation and, consequently, the stress‐related signaling pathways such as JNKs, PKC, GSK3, NF‐kB, and P38 were activated and eventually accelerate insulin resistance.^94^ After the 2.5‐year follow‐up in healthy adults, the obtained results indicated levels of miR‐320a, miR‐122, miR‐197, miR‐15a, miR‐486, and miR‐423 were associated with insulin resistance and response to thiazolidinediones therapy.^95^ In another cohort study on healthy adults, miR‐486‐5p and miR‐320a enhanced the insulin resistance risk. mRNAs of PPAR‐γ and as well, FOXO1, FOXO3 and PTEN are targets of miR‐320a and miR‐486‐5p, respectively, and are associated to insulin metabolism and related conditions such as obesity and dyslipidemia.^96^ Zhang et al reported that low miR‐126 levels might be a predictive marker for the T2DM development in the nondiabetic Han Chinese population. This miRNA is mainly expressed in endothelial cells and contributes to the maintenance of vascular integrity and wound healing. MiR‐126 can modulate the expression of the anti‐inflammatory TOM1 (target of Myb1), vascular endothelial growth factor‐A, and IRS1 genes. Zhang et al speculated that miR‐126 secretion might be induced by insulin and reduced by hyperglycemia.^97^ It has also been suggested that this miRNA modulates expression of various genes that contribute to cholesterol and fatty acid metabolism.^98^ Sansovino et al identified a plasma microRNA signature correlated to retinopathy in T2DM patients. Diabetic retinopathy was associated with increased circulating miR‐25‐3p and miR‐320b levels and decreased miR‐495‐3p levels in T2DM patients compared to diabetic patients without diabetic retinopathy and healthy subjects. Gene ontology analysis demonstrated the enrichment of these targets in signaling pathways such as metabolic processes, stress‐associated cell response, and blood vessel development.^99^ A study conducted by Jones et al showed that circulating levels of miR‐34a, miR‐144‐5p, miR‐532‐5p, and let‐7d closely predicted the insulin resistance phenotype in obese Europeans.^100^ After 4 years of follow‐up, Jiménez‐Lucena et al confirmed that increased levels of miR‐150 and miR‐130a‐5p were related to a decrease insulin sensitivity index and increased level of miR‐375, which are related to decrease in insulin resistance index.^101^ In a meta‐analysis study, Zhu et al suggested that 40 miRNAs were dysregulated in T2DM patients and showed that circulating levels of miR‐29a, miR‐375, miR34a, miR‐103, miR‐132, miR‐107, miR‐142–3p, and miR‐144 may serve as potential T2DM biomarkers.^102^ Sidorkiewicz et al found that miR‐491‐5p, miR‐298, and miR‐1307‐3p can be considered as the diagnostic profiles for predicting the T2DM phenomenon.^103^ The ability of miRNA profiles to predict prediabetic or diabetic conditions should be evaluated in distinct ethnic populations. Specific miRNA profiles may be related to ethnicity‐associated factors. For example, in a Swedish population, miR‐29b, miR‐15a, miR‐126, miR‐144, miR‐24, miR‐191, miR‐486‐5p, and miR‐223 were inversely correlated with insulin sensitivity index, whereas these miRNAs were not correlated with insulin sensitivity index in an Iraqi cohort study, which is suggesting ethnicity.^104^

Recently, studies indicated overexpression of several members of the miR‐141 and miR‐200 family found in the islets of mice lacking the leptin receptor. As well overexpression of various member of these microRNA family correlated with diabetes development. Belgardt et al (2015) indicated that overexpression of these microRNA family members led to elevating the glucose serum level and to development of diabetes in transgenic mice after a few weeks. Belgardt and colleagues generated the mice without expressing the miR‐144 and miR‐200 family members. They found that the transgenic mice were resistant to diabetes.^26^ Furthermore, Takada et al in 2022 illustrated that the paired microRNA including miR‐10a and miR‐200c, or miR‐126 and miR‐10a, was altered in patients with type 2 DM compared to normal subjects (*p* < .05). In their study 50 T2DM patients and 15 normal subjects was involved. The signatures of microRNA serum, miRNA array assessment and also reverse transcription polymerase chain reaction (RT‐qPCR) was analyzed. The miRNA array results revealed that the 19 miRNAs were upregulated over twofold and also 71 miRNAs were downregulated lower than 0.5 in patients with T2DM compared to normal people. Among these miRNAs, four of them was selected to enter the validation analysis with RT‐qPCR based on abundance enough for reliable analyses. Subsequently, in T2DM the serum level of miR‐126‐3p and miR‐10a was up‐ and downregulated, respectively. Their results indicated that the paired‐miRNAs are more effective diagnostics in patients with T2DM.^105^

Yan et al in 2020 study the multiparameter diagnostic model for the early detection of T2DM. In their study miR‐223, miR‐130a, and miR‐19a levels were measured by RT‐qPCR in serum of normal individual and also in impaired glucose regulation and T2DM patients. Finally, their results demonstrated that miR‐223 has the best diagnostic value for distinguishing between impaired glucose regulation and T2DM patients according to receiver operating characteristic (ROC) analysis and the area under the curve (AUC). The AUC of miR‐223 was 0.84. Also, based on the ROC analysis the sensitivity and specificity of miR‐223 was 73.37% and 81.37%, respectively. Additionally, the AUC of four miRNAs was 0.90 and also the sensitivity and specificity were 78.82% and 88.23%, respectively. Moreover, AUC was performed for the validation set and the obtained results indicated that the AUC is 0.88. As well, the sensitivity and specificity for validation set were 78.36% and 87.63%, respectively. Then, their research illustrated that the miR‐148b, miR‐223, miR‐123a, and miR‐19a form the novel multiparameter diagnostic model for the detection of T2DM.^106^

Additionally, Lou et al in 2019 revealed that the miR‐103a and miR‐130b changes have the sensitivity and specificity to differentiate the pre‐DM from T2MD. As well miR‐103a and miR‐103b are the effective biomarkers and high diagnostic value to detection the T2DM. Pre‐DM individuals, noncomplicated diabetic people, and normal glucose‐tolerance individuals (controls) were enrolled. To measure the changes of miR‐103a and miR‐130b they were used bioinformatic analysis, quantitative RT‐PCR, luciferase assays, and ELISA assays.^107^

Moreover, based on the previous studies and according to the role of miR‐34a in lipotoxic effect of pancreatic β‐cells,^108^ hindering Wnt signaling pathway in β‐cells,^109^ increased expression level in peripheral blood mononuclear cells in T2DM patients,^110^ it could be suggested that miR‐34a has the biomarker value to diagnosis and prognosis the T2DM patients. Indeed, based on the several studied on miR‐133, it is illustrated that the upregulation of miR‐133 in patients with prediabetic and diabetes condition, it could be considered as the early diagnostic factor.^111^ In 2020, in the 5‐year prospective study Sidorkiwicz et al indicated that the serum levels of circulating of miR‐491‐5p, miR‐298, and miR‐1307‐3q could be considered as the diagnostic tool for the prediction of T2DM. Their study further suggested that circulating miRNAs are the potential predictive biomarkers of T2DM in prediabetic patients.^103^ Moreover, Sala et al in the study in 2019 revealed that miR‐21 serve as the positive predictive value to early diagnosis of glucose imbalance and also is associated with prediabetic condition.^112^ Further, in 2017 Deng et al was illustrated that the circulating level of miR‐24 may serve as the predictive biomarkers of T2DM and coronary heart disease (CHD). As well, their study suggested that the circulating miR‐24 was able to distinguish between T2DM patients with CHD from CHD patients and controls subjects through an AUC of the ROC of 0.975. Additionally, their suggested that the circulating miR‐24 was not only a potential prognostic value but also a biomarker for predicting DM2 patients with CHD.^113^ As well, in the study by Karolina et al in 2011 revealed that the circulating level of miR‐144a, miR‐146a, miR‐150, miR‐182, miR‐192, miR‐29a, miR‐30d, and miR‐320a serve as special biomarkers that are reflective and predictive of T2DM. Moreover, Al‐Kafaji et al in 2015 suggested that peripheral blood miR‐15a is a potential predictive biomarker of T2DM and prediabetic patients. Their study illustrated that the low level of miR‐15a has the strong association with prediabetes.^114^

## 12 THERAPEUTIC APPROACH OF miRNAs

As previously stated, each miRNA regulates the expression of multiple targets that are frequently associated with the same signaling pathways or functional processes. Thus, therapeutic strategies that modulate the level of specific miRNAs would enable global control of the gene network, in contrast to silencing RNA‐based strategies that target a single gene. This is a significant benefit for therapeutic intervention in the treatment of complex diseases like diabetes.

There are already methods available to alter the level of miRNAs in vivo. Overexpressing miRNAs, also known as miRNA mimics, are short RNA sequences with well‐defined physicochemical properties that can be easily synthesized. These engineered oligonucleotides can be used to raise the degree of cell miRNAs applying a useful impact in sickness settings. miRNAs can likewise be explicitly and effectively inactivated utilizing short antisense oligonucleotides (hostile to miRs) that block their action and advance their debasement.^115^, ^116^ Anti‐miR delivery makes it possible to lessen the impact of miRNAs that contribute to the onset or progression of diabetes. Endogenous RNases found in blood or cells typically have a significant negative impact on RNAs. In any case, miRNA emulates and anti‐miRs can be synthetically altered to expand their dependability and keep away from untimely nuclease corruption.^117^ Chemically modified nucleotides also make it possible to improve target affinity, lessen glomerular filtration, and make it easier for cells to be taken up. Phosphorothioate backbone links are typically present in miRNA mimics and anti‐miRs in order to avoid degradation by nuclease and favor binding to plasma proteins. Additionally, anti‐miRs frequently contain 2′ sugar modifications that enhance complementary RNA affinity and contribute to nuclease resistance.^115^, ^116^ Anti‐miRs have also been conjugated to cholesterol via a 2′‐O‐methyl linkage in some instances to reduce delivery to other tissues and increase hepatic uptake.^116^ Indeed, silencing miR‐103/107 with antisense oligonucleotides enhanced insulin sensitivity and improved glucose homeostasis in either adipocytes or liver.^118^ Also, the Let–7 family of miRNAs enhanced insulin responsiveness in liver and muscle and forestalled impeded glucose resistance in mice with diet‐actuated weight.^119^ Downregulation of miR‐181a and subsequent upregulation of Sirt1, its target, produced comparable outcomes.^120^

Diabetic nephropathy can be alleviated by injecting antisense oligonucleotides that lower miR‐21 levels intraperitoneally.^121^ Mesangial cell hypertrophy, interstitial fibrosis, podocyte loss, and inflammation were among the pathological hallmarks of diabetic kidney disease that were reduced when this miRNA was blocked in diabetic mice. When miR‐192, a transforming growth factor signaling‐induced miRNA, was silenced, similar results were observed.^122^

In addition, diabetic rats treated with intravitreal anti‐miR injections experienced less tissue damage and retinopathy as well as a lower level of miR‐195 in their retinal cells.^123^ In diabetic mice, anti‐miRs have also been used to accelerate wound healing. In fact, in diabetic db/db mice, local administration of anti‐miR‐26 or a miR‐27b mimic was found to accelerate wound healing and promote angiogenesis.^123^, ^124^

It has recently been demonstrated that triantennary Nacetylgalactosamine‐conjugated oligonucleotides, including anti‐miRs, are extremely stable and effectively target the liver.^125^, ^126^, ^127^ In fact, these oligonucleotide conjugates enter hepatocytes via endocytosis after binding to the abundant asialoglycoprotein receptor. The use of N‐acetylgalactosamine conjugates for the delivery of anti‐miRs is currently the subject of clinical trials, and it is highly likely that this strategy will become the most common one for RNA‐based therapies that target the hepatocytes.^127^

To discover the mechanisms that permit cell‐specific uptake, further research into conjugate‐mediated delivery of therapeutic oligonucleotides to other metabolically relevant tissues, such as fat, skeletal muscle, or pancreatic islets, is required. Even though oligonucleotide derivatives have demonstrated efficacy in treating diabetes or the long‐term complications that accompany it, as well as in modulating the level of specific miRNAs, their application has some limitations that have prevented their widespread use for therapeutic purposes.

## 13 EFFECTS OF ANTIDIABETIC DRUGS ON miRNAs

Several miRNAs are associated with the pathophysiology of insulin resistance, T2DM, and promising therapeutic targets have been identified. Antidiabetic drugs have been reported to have beneficial activities in the treatment of insulin resistance and T2DM through miRNA function. Yu et al demonstrated that the pioglitazone/miR‐141/FOXA2 axis is a committed target for T2DM treatment. Pervious study illustrated that FOXA2 is an important regulator in development of β‐cells and insulin secretion. miR‐141 upregulation caused impaired glucose‐mediated insulin secretion and pancreatic β‐cells proliferation by targeting FOXA2. Pioglitazone is an agonist of PPAR‐γ and an antidiabetic drug extensively used to enhance insulin production and sensitivity. Yu et al showed that miR‐141 expression was corrected with pioglitazone treatment.^128^ Furthermore, pioglitazone regulate the macrophage polarization via PPAR‐γ/miR‐223 axis. miR‐223 axis, which is an important regulator of macrophage polarization, adipose tissue‐associated inflammation, and insulin resistance. Basically, PPAR‐γ regulates the miR‐233 expression through targeted the miR‐233 promoter elements.^129^

Metformin, an AMPK activator, is another widely used antidiabetic agent to improve insulin sensitivity and decreasing blood glucose in T2DM patients. AMPK activation by metformin augmented the level of the miRNA‐processing enzyme DICER. It overexpressed levels of miR‐30b and miR‐30c in hepatocytes, and coupling to increased mitochondrial function and carnitine palmitoyl transferase 1 α expression, leading to decrease lipogenesis and increased fatty acid oxidation.^130^ miR‐291b‐3p/AMPK axis, which inhibits hepatic lipogenesis through AMPK activation and miR‐291b‐3p downregulation in mice, is another mechanism through metformin exerts its impacts.^131^ In another study conducted by Zheng et al revealed that metformin overexpressed miR‐185–5p level in vitro and in vivo, thereby preventing the activity of glucose‐6‐phosphatase alleviating hepatic gluconeogenesis and subsequently fasting hyperglycemia. Mechanistically, miR‐185–5p suppresses hepatic glucose output by directly binding to the 3′UTR region of glucose‐6‐phosphatase sequence.^132^ In a recent study, Naghiaee et al represented that metformin treatment embeds insulin resistance in human diabetic adipose tissue by suppressing miR‐223 expression. This miRNA targets the IRS1/Akt/GLUT4 signaling pathway and plays a critical role in the onset of T2DM.^133^

Skeletal muscle is one of the main targets of insulin. Some studies have highlighted metformin's effect in modulating glucose metabolism in skeletal muscle, and some miRNAs have been implicated in the regulation of glucose metabolism in this target tissue. For example, miR‐21 is one of the critical miRNAs involved in physiological processes in skeletal muscle. Wang et al showed that metformin reducedmiR‐21 expression and this was associated with a reduction in insulin resistance. They introduced Smad7 as an effective target of miR‐21. Their results suggests that metformin reduces insulin resistance in skeletal muscle via upregulation of Smad7 expression, a target of miR‐21.^134^ Moreover, Demirsoy et al confirmed a circulating miRNA profile following metformin therapy in T2DM patients. They observed the serum levels of 13 candidate miRNAs (miR‐let‐7e‐5p, let‐7f‐5p, miR‐21‐5p, miR‐24‐3p, miR‐26b‐5p, miR‐126‐5p, miR‐129‐5p, miR‐130b‐3p, miR‐146a‐5p, miR‐148a‐3p, miR‐152‐3p, miR‐194‐5p, miR‐99a‐5p) were significantly decreased after treatment by metformin in T2DM patients.^135^ In a randomized, placebo‐controlled, and double‐blind validation study, Ortega et al reported four circulating miRNAs (miR‐140‐5p, miR‐142‐3p, miR‐192, and miR‐222) that were markedly altered in parallel with decreased fasting glucose levels after metformin treatment in T2DM patients. Their results revealed that miR‐140‐5p and miR‐222 were decreased and miR‐142‐3p and miR‐192 were increased in metformin‐treated T2DM patients.^136^ Sansovino et al conducted an experimental study inT2DM patients through 12 months of metformin treatment. They revealed that metformin increased let‐7a expression and did not alter miR‐326 expression.^137^

## 14 EFFECTS OF POLYPHENOLS ON microRNAs

In addition, polyphenols such as curcumin, epigallocatechin gallate (EGCG), resveratrol, and quercetin can also modulate miRNAs for the therapy of diabetic complications.^132^, ^134^, ^138^ In a previous study, Liu and co‐workers showed that quercetin and EGCG improved insulin resistance and hepatic gluconeogenesis via the IRS1/Akt/FOXO1 axis and involving miR‐27a‐3p and miR‐96–5p. FOXO1 is identified as a direct target of miR‐27a‐3p and miR‐96–5p and exerts its protective effect through the downregulation of PEPCK and glucose6‐phosphatase.^139^ In another study, Liu et al also reported the synergistic protection effect of EGCG and quercetin against streptozotocin‐mediated pancreatic β‐cell damage via downregulation of miR‐16‐5p expression that directly targets the Bcl‐2 gene.^140^ Numerous studies have demonstrated that resveratrol mediates anti‐inflammatory activities against patients with T2DM. The results provided by Tomé‐Carneiro et al. found that supplementation with a grape extract containing resveratrol modulates inflammation‐related miRs (miR‐155, miR‐21, miR‐181b, and miR‐34a) and pro‐inflammatory cytokines (CCL3, IL‐1β and TNF‐α) in peripheral blood mononuclear cells of patients with T2DM. These miRs are associated with inflammation and contribute to the regulation of Toll‐like receptors, NF‐κB signaling, and the levels of inflammatory cytokines such as IL‐1β and TNF‐α.^141^ Mahjabeen et al showed a protective role of resveratrol supplementation against inflammation and oxidative stress in T2DM patients. Resveratrol supplementation at 24 weeks resulted in significant downregulation of miRNA‐34a, miRNA‐375, miRNA‐21, and miRNA‐192, and upregulation of miRNA‐126 and miRNA‐132. Downregulation of miRNA‐34a effectively resulted in the upregulation of Sirt1, a putative mechanism responsible for resveratrol's reduction of oxidative stress and inflammation in T2DM. Besides, upregulation of miRNA‐132 could suppress inflammatory responses mediated by NF‐kB, TNF‐α, and toll‐like receptor pathways.^142^ Moghassemi et al. have been shown raspberry fruit extracts decrease serum levels of malondialdehyde in diabetic Wistar rats compared to healthy controls.^143^ Recent studies have revealed that berberine can improve hepatic glycogen synthesis by modulating the Sirt1/FOXO1 pathway. Sirt1 plays a positive role in metabolic disorders such as obesity and T2DM through deacetylating FOXO1 to alleviate oxidative stress. Sui et al found that berberine could improve hepatic insulin sensitivity by targeting the miR‐146b/Sirt1/FOXO1 axis. Their results suggested that miR‐146b directly targeted the Sirt1 gene and enhanced acetylate‐FOXO1 activity in HepG2 cells. Berberine's hepatoprotective effects may through suppression of miR‐146b expression, resulting in Sirt1/FOXO1 expression.^83^ These studies suggested that polyphenols could control T2DM by regulating various miRs, which could represent a potential therapeutic strategy to improve T2DM.

## 15 CONCLUSIONS

This review article summarizes the changes in miRNAs involved in beta cell function, insulin secretion, glucose tolerance, and insulin function in obesity, inflammation, and insulin resistance states. It has been suggested that all the metabolic changes are associated with an increased risk of developing T2DM. Therefore, the described miRNAs could illustrate a potential molecular link between the metabolic changes associated with obesity, inflammation, and the onset and development of T2DM However, based on the knowledge gap on miRNAs and their role in the pathogenesis and treatment of T2DM, further exploration of their involvement in different stages of T2DM is necessary.

## FUNDING INFORMATION

This work was supported by Golestan University of Medical Sciences (grant agreement number IR.GOUMS.REC.1399.229).

## CONFLICT OF INTEREST STATEMENT

The authors declare that they have no conflict of interest.

## ETHICS STATEMENT

All of this work was approved by ethics committee of Golestan University of Medical Sciences (grant agreement number IR.GOUMS.REC.1399.229).

## Data Availability

The authors confirm that the data supporting the findings of this study are available within the article and its supplementary materials.
